# Neurologic improvement and tumor shrinkage after radiotherapy in dogs with imaging-based intracranial neoplasia

**DOI:** 10.1093/jvimsj/aalag069

**Published:** 2026-04-27

**Authors:** Nina Ruessli, Robert Herzig, Chris Staudinger, Felicitas Czichon, Valeria Meier, Richard Evans, Katrin Beckmann, Carla Rohrer Bley

**Affiliations:** Clinic for Radiation Oncology & Medical Oncology, Small Animal Department, Vetsuisse Faculty, University of Zurich, Zurich, Switzerland; Division of Small Animal Neurology, Small Animal Department, Vetsuisse Faculty, University of Zurich, Zurich, Switzerland; Clinic for Diagnostic Imaging, Department of Clinical Diagnostics and Services, Vetsuisse Faculty, University of Zurich, Zurich, Switzerland; Clinic for Radiation Oncology & Medical Oncology, Small Animal Department, Vetsuisse Faculty, University of Zurich, Zurich, Switzerland; Clinic for Radiation Oncology & Medical Oncology, Small Animal Department, Vetsuisse Faculty, University of Zurich, Zurich, Switzerland; Masonic Cancer Center, University of Minnesota, Minneapolis, MN, United States; Division of Small Animal Neurology, Small Animal Department, Vetsuisse Faculty, University of Zurich, Zurich, Switzerland; Clinic for Radiation Oncology & Medical Oncology, Small Animal Department, Vetsuisse Faculty, University of Zurich, Zurich, Switzerland

**Keywords:** brain tumor, glioma, IMRT, meningioma, neurodisability score, pituitary tumor

## Abstract

**Background:**

Traditional measures of treatment success for radiotherapy in dogs with intracranial neoplasia include progression-free and overall survival time. Although important, these measures do not reflect neurologic function.

**Hypothesis/Objectives:**

Assess tumor shrinkage by follow-up imaging and outcome using 2 neurodisability scoring systems—1 validated and 1 simplified.

**Animals:**

One hundred six dogs with imaging-diagnosed intracranial tumors treated with 10-fraction definitive-intent radiotherapy.

**Methods:**

Data were collected from 2 randomized trials. Neurologic function was prospectively assessed using a validated score, and a retrospective simplified score was added. Imaging was recommended every 6 months or upon clinical decline.

**Results:**

Diagnoses included extraparenchymal tumors (45.3%), intraparenchymal tumors (35.8%), and pituitary tumors (18.9%). Median follow-up was 581 days. The neurodisability score improved significantly before radiotherapy (median 1.0, *P* = .04) because of medical management, and again during treatment (median 0.0, *P* < .01). At peak response, 76% of dogs had no or only mild neurologic deficits. Tumor volume significantly decreased at 6 and 12 months (*P* < .01): median shrinkage at 6 months was −39% (extraparenchymal tumors), −83% (intraparenchymal tumors), and −47% (pituitary tumors). A moderate correlation between tumor reduction and neurodisability score was seen only at 6 months (*r* = 0.395, *P* = .002). Results were consistent across protocols.

**Conclusions and clinical importance:**

Radiotherapy led to lasting neurologic improvement and substantial tumor reduction. Neurologic function did not always correlate with tumor volume shrinkage, emphasizing the importance of incorporating and prioritizing neurologists’ functional assessments in posttreatment evaluation.

## Introduction

Outcomes after conventionally fractionated radiotherapy for brain tumors in dogs are usually reported as time to progression, progression-free, or overall survival.^1–3^ These measures do not reflect the dog’s clinical state (ie, neurologic well-being) after treatment. Commonly, the severity of neurologic signs is not only a result of tumor size but also of location.^1^^,^^4^

After radiotherapy, tumors usually shrink in size.^2^^,^^5^ Shrinkage as well as time to smallest size vary, and are not well described. In imaging-based follow-up examinations, tumor volume changes after radiotherapy can be quantified using linear measurements, volumetric analysis, or more comprehensively assessed with standardized response criteria such as the Macdonald criteria, the response assessment in neuro-oncology, or the response evaluation criteria in solid tumors framework.^6^^,^^7^ The goal of therapy—clinically improved neurologic status—is achieved by reduction in tumor burden and alleviation of pressure on surrounding neural structures. In veterinary medicine, however, these neurologic improvements are at most reported as limited and unstructured information in addition to classical oncologic responses such as progression-free and survival outcomes. Most dogs (75%-100%) show subjective neurologic improvement after radiotherapy, with improvements typically beginning during or shortly after treatment.^1^^,^^3^^,^^5^ However, this improvement is mostly based on information provided by owners or documented by the treating radiation oncologist and usually lacks systematic assessment using a defined scoring system, along with the expertise of a neurologist.^8–11^

A neurologic scoring system originally developed to evaluate meningoencephalomyelitis of unknown etiology has previously been applied to quantify the severity and progression of neurologic dysfunction in affected dogs.^12^ As a standardized assessment tool, this system also has been utilized in dogs diagnosed with intracranial neoplasia.^3^ Although comprehensive in its individual components, the scoring system does not provide an integrated measure of overall neurologic well-being. Furthermore, tumor shrinkage has never been associated with the course of dogs’ neurologic signs. Repeated volumetric follow-up after radiotherapy in intracranial neoplasia is seldom performed in a standardized manner, because of financial considerations and the lack of therapeutic consequences in a clinically normal animal. On clinical deterioration, dogs with brain tumors usually are reassessed using diagnostic imaging. Without regular follow-up imaging, it remains unclear at which point the smallest tumor volume is reached.

Herein, we describe the severity of neurologic dysfunction and its course after treatment, including 2 datasets of dogs treated with radiotherapy in randomized controlled clinical trials. We used an established scoring system^3^^,^^12–15^ and a more general neurologic classification of severity of neurologic dysfunction (mild, moderate, severe signs) in parallel with imaging-based volumetric tumor measurements during a follow-up period after radiation therapy. The following questions were addressed: (1) Can we describe a general timeline of neurologic improvement, and (2) are clinical signs associated with tumor size? We hypothesized that neurologic signs would resolve rapidly in most dogs and that the severity of clinical signs would correlate with tumor size.

## Material and methods

### Study design and inclusion criteria

Our dataset was derived from 2 single-center, prospective, randomized, controlled, parallel-group studies conducted at the Clinic for Radiation Oncology & Medical Oncology of the Vetsuisse Faculty, University of Zurich, Switzerland.^3^ The study included dogs of all breeds, ages, and sexes that were referred for radiotherapy, presenting with neurologic signs and an imaging-based diagnosis of primary intracranial neoplasia. [Raw data available in open repository: https://dataverse.harvard.edu/dataset.xhtml?persistentId=doi:10.7910/DVN/HZ6ZFJ].

Eligibility criteria required that the dogs had no evidence of metastasis and had not undergone prior treatment with surgery, radiation therapy, or chemotherapy. Owner consent was mandatory, and both studies were approved by the Animal Ethics Council of the Canton of Zurich, Switzerland (Permit Numbers: ZH075/17, ZH021/19, and ZH202/2022).

Initial evaluation included a history and physical examination, documentation of corticosteroid and other medical treatment use and dose, and brain imaging. Before treatment, a resident or diplomate of the European College of Veterinary Neurology assessed each dog’s neurologic status using a published neurodisability scoring system (File S1).^12^

In addition, neurologic deficits were scored using an adapted classification:^9^^,^^16^

Mild = mild neurologic deficits, including head tilt or proprioceptive deficits only, whereas the animal remains alert and ambulatory; may also include focal or isolated seizures.

Moderate = noticeable neurologic dysfunction in an ambulatory animal without a decreased level of consciousness, encompassing paresis, vestibular or cerebellar ataxia, hypermetria, cranial nerve deficits, nystagmus, or cluster seizures that can be managed at home.

Severe = debilitating neurologic deficits, such as ataxia with leaning or falling, nonambulatory paresis, dysphagia, circling, decreased mentation, or status epilepticus or cluster seizures necessitating hospitalization.

Tumors were categorized into 3 groups based on imaging characteristics: extraparenchymal tumors (all extraparenchymal, non-pituitary tumors, including tumors in the cerebellar pontine angle, but not within the lateral or the third ventricle), intraparenchymal tumors (all intraparenchymal, non-pituitary tumors), and pituitary tumors.

### Medical and radiation therapy

Medical treatment before and after radiotherapy was not standardized but tailored to the specific needs of each dog. Medication was typically initiated on the day of diagnosis or when neurologic signs first appeared and was adjusted based on improvement of clinical signs. Treatment mainly involved corticosteroids and anticonvulsant drugs in dogs with seizures.

Dogs were assigned to receive intensity-modulated radiotherapy (IMRT) in 10 fractions, with or without boost. Boost treatment refers to the delivery of an additional, focused radiation dose to the bulk of the tumor to increase local tumor control while minimizing exposure to surrounding normal tissues. Treatment procedure adhered to strict protocols from 2 prospective randomized trials and has been described in detail elsewhere.^3^^,^^17^ In brief, the gross tumor volume (GTV) was defined and contoured using contrast-enhanced computed tomography (CT) or magnetic resonance imaging (MRI), and in tumors with no contrast uptake, T2 sequences were used.^18^ The clinical tumor volume accounted for subclinical disease extension and was expanded by 2 mm to define the planning target volume. Organs at risk were segmented as previously described. Treatment planning followed the recommendations for IMRT.^19^ Dose was either prescribed homogeneously (10 × 4 Gy) or in 10 fractions with a simultaneously integrated boost or a heterogeneous boost.^3^^,^^17^ All dogs were treated using a Varian Clinac iX 6MV linear accelerator and intensity-modulated treatment (IMRT or volumetric modulated arc therapy), with daily positioning verification. Treatment was delivered in 10 fractions, daily over 2 weeks.

### Assessment after therapy and follow-up

Clinical assessment after therapy was encouraged 3 weeks after radiotherapy, at 3 months, and then every 6 months until progression or death. Neurologic examination, classification, and neurodisability score assessment, as well as documentation of corticosteroid and other medical treatment use and dosage, followed the same schedule. Diagnostic imaging was recommended every 6 months or at the time of deterioration of clinical signs. If the owners were unavailable in person, follow-up was conducted via telephone or email.

Reevaluation MRI and CT scans were imported into Eclipse External Beam Planning software, and any tumor remnants were segmented on the co-registered images from the initial scan. Volumetric measurements were taken, and relative change in size compared to the initial GTV was calculated.

Determination of progression was at the clinical team’s discretion, which included neurologic examination with or without diagnostic imaging or findings at necropsy. Progressive disease was considered if neurologic signs worsened, were neither transient nor corticosteroid-responsive, or if the tumor volumetrically increased (ie, any increase in enhancing tumor or T2W/FLAIR lesion burden, or a new, metastatic lesion).^20^^,^^21^ We also reported separately any volumetric increase of ≥ 40%, extrapolated based on volumetric criteria proposed in the literature relative to the last measurement.^21^

### Statistical analysis

Statistical analysis was performed in 3 parts: a preprocessing part, descriptive part, and an inferential part. In the preprocessing part, counts, percentages, means, standard errors, medians, and interquartile ranges (IQRs), as well as histograms were used to assess variable distributions, and inspect for spurious observations. Missing data were handled using the available case approach. For the descriptive part, counts of dogs (with percentages) were used to describe the patterns of change in neurodisability scores and tumor sizes in a longitudinal fashion. In addition, spaghetti plots were used to visualize within-subject trends. For the inferential part (which provides *P*-values), the general approach for the longitudinal data was to use robust, nonparametric pairwise statistical tests rather than longitudinal models with possibly difficult-to-verify assumptions. Fisher’s exact test was used to compare counts of dogs that did or did not change (neurodisability score, tumor size) over pairs of time points. For non-longitudinal covariates (eg, tumor type), Kruskal–Wallis analysis of variance (ANOVA) followed by Tukey-adjusted pairwise tests were used. The correlation between neurodisability score and tumor size was assessed using Spearman’s correlation. Following the convention for controlling “family-wise error rates,” *P*-values within similar types of hypotheses were adjusted for multiplicity (eg, following nonparametric ANOVA) but others were not (eg, the correlation test *P*-value). Statistical significance for adjusted and unadjusted *P*-values was .05.

## Results

### Dog and tumor characteristics

A total of 106 dogs completed treatment between September 2017 and July 2023 and were followed until January 2025. File S1 summarizes details concerning dogs, tumors, and neurologic signs. The most common lesions based on imaging were extraparenchymal tumors (48/106, 45.3%), followed by intraparenchymal tumors (38/106, 35.8%), and pituitary tumors (20/106, 18.9%). Most tumors (87/106, 82.1%) were located rostrotentorially. Based on the radiology reports of the included dogs, the primary suspected diagnosis for all included extraparenchymal tumors was meningioma, whereas the primary suspected diagnosis for all intraparenchymal tumors was glioma. Extraparenchymal lesions accounted for the majority of caudotentorial tumors (17/19, 89%), whereas only 2 (2/19, 11%) imaging-based intraparenchymal tumors were present in this location. Before treatment, tumors in the rostrotentorial region were significantly larger than tumors in the caudotentorial region (*P* = .01). The volumetric median for rostrotentorial tumors before treatment was 3.22 cm^3^ (IQR = 0.17%-11.84%; *n* = 87), whereas median measurements for caudotentorial tumors were 2.30 cm^3^ (IQR = 0.90-6.90; *n* = 19). Seizures were the most common neurologic sign in dogs with extraparenchymal tumors (23/48, 48%) and intraparenchymal tumors (34/38, 89%), whereas all dogs with pituitary tumors exhibited abnormal mental status (20/20, 100%). Ninety-four dogs (88.7%) had MRI before treatment. In the other 12 dogs (9 extraparenchymal tumors, 3 pituitary tumors), the diagnosis was made by CT. Otherwise, the 2 groups were balanced concerning baseline variables (File S1). Neither dog nor tumor characteristics, tumor shrinkage, neurologic presentation, nor outcome differed between protocols; therefore, results were not further divided by protocol type.

### Neurologic assessment: neurodisability scores

Throughout treatment and follow-up, we repeatedly assessed the 106 dogs using the neurodisability score, resulting in 544 individual scores. Forty dogs (37.7%) were initially diagnosed at our clinic and had a score recorded at the time of tumor diagnosis. On the first day of radiotherapy, 99 of 106 dogs (93.4%) underwent complete neurologic examination and were scored before treatment. In a few cases, neurologic assessment could not be performed because of the dogs’ uncooperative behavior or scheduling conflicts that prevented a neurologist from being available.

**Figure 1:**
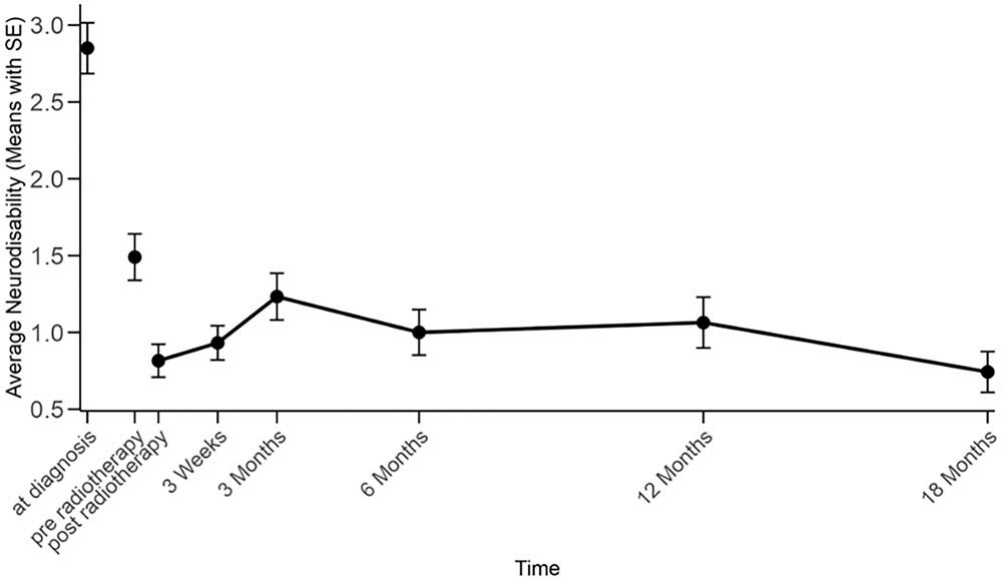
Average neurodisability score over time, display of mean scores: the largest change was seen from diagnosis to treatment start (effect of initial medical support) and during radiotherapy.

On the final day of treatment, 103 of 106 dogs (97.2%) were re-evaluated. At the time of progression (in 59 dogs overall), scoring was possible in 30 dogs (51%). The average neurodisability scores are presented in File S1. Figure 1 illustrates the course of mean neurodisability scores over time. The largest improvement of neurodisability score occurred between diagnosis and radiotherapy initiation (median score, 1.0; *P* = .04), likely because of medical interventions such as corticosteroid administration and anticonvulsant treatment. Another meaningful improvement was observed from the beginning to the end of radiotherapy (median score, 0.0; *P* < .001). Between 12 and 18 months posttreatment, 33% (9/27) of the dogs showed some clinical decline with an increase in their mean neurodisability score, 56% (15/27) remained stable, and 11% (3/27) demonstrated further improvement. At diagnosis, 25 of 106 dogs (23.6%) had a known concurrent spinal disease unrelated to their intracranial tumor, with 44% of these cases occurring in brachycephalic breeds.

### Neurologic assessment: severity of neurologic dysfunction

**Figure 2:**
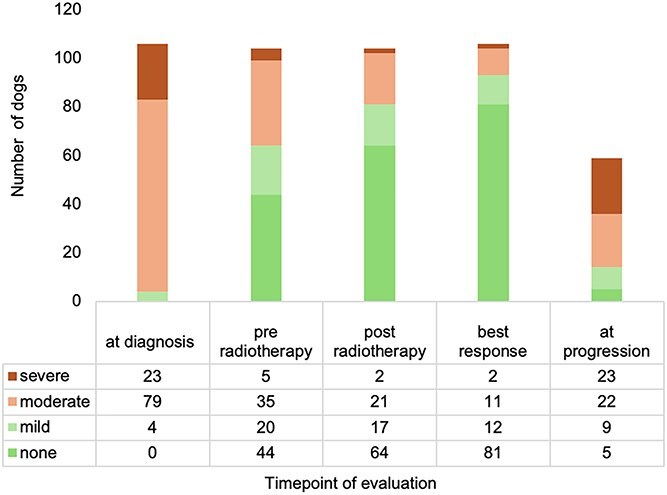
Severity of neurologic dysfunction (neurologic deficit classification) and number of dogs at the respective time points.

Neurologic dysfunction was also repeatedly scored over time. All dogs presented with marked neurologic deficits at diagnosis: 4% with mild, 75% with moderate, and 21% with severe signs (Figure 2).

With medical treatment, neurologic signs were significantly and strongly decreased in most dogs even before the initiation of radiotherapy (*P* = .003). Further improvement in neurologic function was observed from the first to the last day of radiotherapy (*P* = .003). By the end of radiotherapy, only 40 of 104 dogs (38.5%) exhibited residual neurologic abnormalities, of which 16.3% (17/104) were classified as mild, 20.2% (21/104) as moderate, and 1.9% (2/104) as severe. At the individual time point of best neurologic response, only 25 of the 106 dogs (23.6%) showed any remaining neurologic deficits. Of these, 2 (8%) exhibited severe, 11 (44%) moderate, and 12 (48%) mild neurologic deficits.

At the time of clinical progression, neurologic dysfunction scores were available for all 59 dogs. Of these, 23 (39%) exhibited severe, 22 (37%) moderate, and 9 (15%) mild neurologic deficits. Five dogs (9%) remained asymptomatic despite radiologic evidence of tumor progression.

### Neurologic assessment: seizures

Of the 58 dogs that initially presented with epileptic seizures, 25 had extraparenchymal tumors, 32 intraparenchymal tumors, and 1 had a pituitary tumor. Thirty-one (53%) remained seizure-free after radiotherapy. Among dogs with recurrent seizures (*n* = 27), seizure activity was observed exclusively at the time of tumor progression in the majority of cases (18/27; 67%). Median time to seizure recurrence in dogs that experienced seizure control after radiotherapy was 12 months (IQR = 3-30 months; *n* = 18). A small proportion of dogs (5/58; 9%), all diagnosed with extraparenchymal tumors, showed a delay in achieving freedom from seizures and experienced seizures within the first 3 months after radiotherapy only. In 4 dogs (4/58; 7%) seizures occurred beyond the initial 3-month period in the absence of suspected or confirmed tumor progression (3 with extraparenchymal tumors;1 with an intraparenchymal tumor). In 2 of these 4 dogs (2/58; 3%; both with extraparenchymal tumors), adequate seizure control was not achieved at any point during follow-up. Medical treatment is described below.

### Follow-up and disease progression

Median follow-up time was 581 days (range, 45-1893 days). At the conclusion of the study, 18 of 106 dogs (16.9%) were still alive, and 9 dogs (8.4%) had been lost to follow-up and therefore were censored at the date of last available information.

Overall, 85 dogs (80.2%) underwent at least 1 follow-up imaging examination after radiation therapy, resulting in a total of 172 imaging studies. Of these, 141 (82%) were conducted as routine reevaluations at fixed time points. Follow-up MRIs were performed as follows: 28 dogs had 1 MRI, 23 had 2, 11 had 3, 10 had 4, and 1 dog underwent 5 MRIs. Follow-up CT scans were performed once in 11 dogs, twice in 3 dogs, and 3 or 4 times in 1 dog each. Magnetic resonance imaging was recommended for follow-up imaging. In dogs with suspected disease progression, CT was offered as an alternative for extraparenchymal tumors when owners declined MRI. In some cases, follow-up imaging was performed at external institutions (44 MRIs and 4 CTs), where the choice of imaging modality was determined by the attending clinicians.

**Figure 3:**
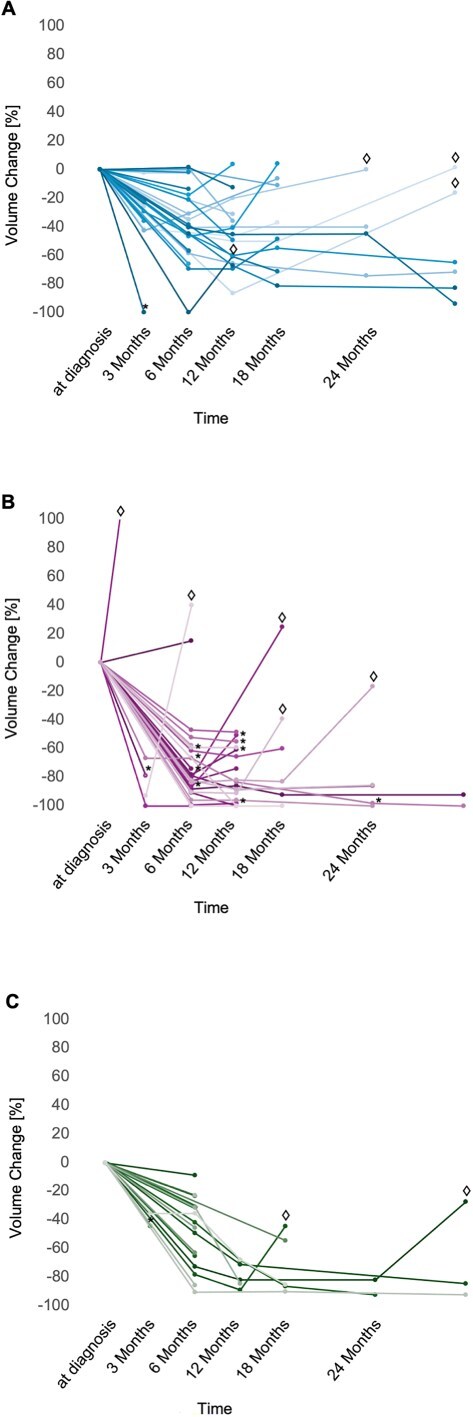
(A-C) Changes in relative tumor volumes after treatment: (A) extraparenchymal tumors (clinically diagnosed meningiomas), (B) intraparenchymal tumors (clinically diagnosed gliomas), and (C) pituitary tumors. Each line represents an individual dog with follow-up imaging, while each dot represents a time point of imaging. The baseline value represents the tumor volume at diagnosis. The connecting lines are for better visualization only and do not reflect a linear volume change between the imaging time points. The symbol ♢ indicates ≥ 40% volume increase relative to the smallest volume measured, while the * symbolizes concurrent metastasis.

During the follow-up period, 59 of 106 dogs (55.7%) were considered to have progressive disease (worsening neurologic signs neither transient nor corticosteroid-responsive, any volumetric increase in the target lesion, or the appearance of new metastatic lesions). Among these, 54 dogs (92%) exhibited clinical deterioration. Twenty-three dogs (39%) were classified as having progressive disease based solely on clinical signs, whereas 31 dogs (53%) underwent imaging that confirmed that clinical deterioration was associated with an increase in tumor volume Figure 3. In 5 additional cases (8%), a volumetric increase was detected incidentally during routine follow-up imaging. In one of these dogs, metastasis was diagnosed, whereas in the 4 remaining cases, volumetric progression with a median 41.2% increase (IQR = 40%-44.2%; *n* = 4) was found. The previously described cut-off of > 40%^20^^,^^21^ increase in volume was only achieved in 11% of dogs (19%), all of which showed neurologic deterioration at the time of imaging. In 12 dogs (20%), central nervous system metastasis was part of the progression pattern; all dogs also showed neurologic deterioration at this point: 10 dogs with intraparenchymal tumors, 1 with an extraparenchymal tumor, and 1 with a pituitary tumor. In all but one of these dogs (11/12), the primary disease was either in complete (*n* = 3) or partial (*n* = 8) remission. In the remaining dog, imaging was performed at another clinic. Although metastasis could be confirmed based on the provided images, assessment of the volumetric changes was not possible because of technical issues.

In 72% of dogs that experienced renewed tumor growth, the tumor volume did not exceed the original size at diagnosis. Complete tumor regression was documented in 10 of the 106 dogs (9%), consisting of 8 with intraparenchymal tumors and 2 with extraparenchymal tumors.

Compared to baseline measurements, tumor volume reductions were observed as follows: at 6 months, tumor volumes had decreased by a median of −58% (IQR = −83%-−35%; *n* = 68). At 12 months, the reduction was a median of −66% (IQR = −86%-−48%; *n* = 40). At 24 months, sustained volume reductions were seen in 8 dogs, with a median of −82% (IQR = −92%-−40%; *n* = 8). Beyond 30 months, sustained volume reductions were seen in 8 dogs, with a median of 83% (IQR = −92%-−27%; *n* = 8).

At the 6-month time point, tumor volume reductions differed significantly by location (*P* = .01), with tumors in the rostrotentorial region showing a larger median decrease of −63% (IQR = −86%-−40%; *n* = 61), compared with caudotentorial tumors, which showed a median reduction of −33% (IQR = −45%-−2%; *n* = 10). At all subsequent time points, no significant differences in volume change were observed between these 2 anatomical locations.

When analyzed by tumor type, significant volume reductions were observed at both 6 and 12 months (*P* < .001 for both time points; Figure 3). At 6 months, median tumor shrinkage was −39% for extraparenchymal tumors (IQR = −47%-−14%; *n* = 27), −83% for intraparenchymal tumors (IQR = −92%-−70%; *n* = 28), and −47% for pituitary tumors (IQR = −75%-−31%; *n* = 16). At 12 months, the reductions remained significant, with extraparenchymal tumors showing a median decrease of −48% (IQR = −61%-−36%; *n* = 18), intraparenchymal tumors −83% (IQR = −96%-−59%; *n* = 19), and pituitary tumors −76% (IQR = −84%-−68%; *n* = 6).

A moderate positive correlation (*r* = 0.395, *P* = .002) was observed between neurodisability scores and tumor volume reduction at 6 months. No significant correlations were found at any other time point. Details on oncologic outcomes, including time-to-progression and survival, are reported elsewhere.^3^

### Corticosteroids and anticonvulsant use

All but one dog (105/106, 99.1%) received prednisolone at some point during treatment. At diagnosis, the majority (95/106, 89.6%) began prednisolone with an initial average daily dosage of 0.83 mg/kg PO (IQR = 0.5-1.0; *n* = 101). This dosage was tapered to 0.6 mg/kg PO (IQR = 0.5-1.0; *n* = 104) before radiotherapy and further decreased to 0.55 mg/kg PO (IQR = 0.45-0.75; *n* = 104) by the end of radiotherapy. Prednisolone tapering was individualized based on neurologic status. By 3 months after radiotherapy, the median dosage had decreased to 0.18 mg/kg PO (IQR = 0.0-0.38; *n* = 94), and by 6 months, most dogs were no longer receiving corticosteroids (median dosage, 0 mg/kg PO; IQR = 0.0-0.16; *n* = 84).

In 13 dogs (13/101, 12.9%), early-delayed radiation toxicity was suspected and managed with a temporary increase in corticosteroid dosage. Beyond 6 months, the median corticosteroid dosage remained at 0 mg/kg, but some dogs required reintroduction of corticosteroids because of neurologic decline. From 3 months onward, tumor progression accounted for most increases in prednisolone use (25/27 cases, 92%). No significant differences in prednisolone administration were observed between treatment protocols (*P* > .3).

All dogs presenting with seizures (58/58, 100%) received concurrent anticonvulsant treatment, either as monotherapy or in combination, consisting primarily of phenobarbital and levetiracetam. No attempt was made to completely wean off anticonvulsant treatment, and treatment was continued after radiotherapy in all dogs. Dosage was tailored based on individual clinical response.

## Discussion

Neurologic status is a key indicator of well-being in dogs after radiotherapy. In our study, the neurodisability score showed only a weak to moderate correlation with imaging-based tumor volumes, and only at the 6-month time point. Both the neurodisability score and changes in the severity of neurologic dysfunction improved rapidly with initial medical management and continued to improve during radiotherapy, with effects that were largely sustained over time. Volumetric tumor regression was substantial and varied in magnitude and timing depending on tumor type. Notably, recurring clinical deterioration often was accompanied by only mild increases in tumor volume, frequently below the commonly used ≥ 40% volumetric progression threshold, and hence often still below baseline measurements.^20^^,^^21^

Rapid neurologic improvement achieved with medical management is often beneficial to the stabilization of dogs before starting radiotherapy. Medical management, specifically with corticosteroids, was found to objectively decrease peritumor brain edema in approximately 50%-60% of gliomas and meningiomas and was significantly linked to larger peritumoral edema and mass effect reduction. However, corticosteroids also appeared to provide clinical benefits to dogs without a measurable decrease in edema.^22^ We observed a significant decrease in neurological signs in most dogs before the start of radiation therapy, particularly after corticosteroid administration. The substantial improvement in quality of life observed by owners in their dogs after the initiation of radiotherapy is likely attributable to corticosteroid treatment and not radiation therapy, especially because this improvement often occurs within the first 3 weeks of treatment, and even in cases in which a measurable reduction in tumor volume was not yet apparent at the time of initial reimaging.^2^ The rapid clinical response observed early in the treatment course is therefore consistent with a corticosteroid-mediated decrease in peritumoral edema and intracranial pressure, rather than an immediate radiotherapy-induced effect on tumor volume.

We do not, however, consider medical management alone as a therapeutic option. Although palliative medical management can be offered to clients who do not want to pursue radiotherapy or surgery, it only improves neurologic signs for a few weeks.^9^^,^^23^

Tumor volume reduction at 6-month follow-up varied by tumor type, with intraparenchymal tumors demonstrating the earliest and most pronounced shrinkage (83%), whereas extraparenchymal tumors and pituitary tumors exhibited slower but more sustained decreases in size (39% and 47%, respectively). Quantitative assessments of tumor volume reduction in brain tumors in dogs after radiation therapy are limited. Rapid tumor shrinkage was also observed in intraparenchymal tumors after radiotherapy, with a mean decrease in tumor size of 75% between diagnosis and the first reevaluation noted 3 months posttreatment.^2^ In an evaluation of macroadenomas treated by radiotherapy, repeated brain imaging was performed in a few dogs at various time points. Although only a few repeated images were available, substantial and sustainable tumor shrinkage was reported.^24^

Tumors in the caudotentorial region initially were smaller than those in the rostrotentorial region. In the caudotentorial region, tumors are commonly associated with earlier onset and more severe neurological deficits because of the vital function of the brainstem and are therefore diagnosed at an earlier stage.^1^^,^^16^ At 6 months after radiotherapy, tumors in the rostrotentorial region were significantly smaller than tumors in the caudotentorial region. Tumor type is not distributed equally among locations. In the caudotentorial regions, extraparenchymal tumors are much more likely than intraparenchymal tumors.^25^ Therefore, this difference is most likely because of tumor type and not location.

In extraparenchymal tumors, we observed a median volume reduction of 39% after 6 months, while clinically neurologic signs remained absent or minimal. This observation emphasizes that volume reduction need not be massive to achieve sustainable neurologic improvement. On the other hand, marked shrinkage of the primary tumor does not guarantee a favorable outcome, because metastases still may be present despite substantial tumor shrinkage.^26^ These findings suggest that imaging-based volume shrinkage alone does not fully reflect the neurologic well-being of affected dogs, emphasizing the importance of incorporating clinical and quality-of-life assessments along with imaging.

Neurologic examination might well be the most dependable assessment: The neurodisability score proved effective for monitoring neurologic status in individual dogs, independently of tumor volume metrics. Although it requires in-person evaluation by a trained neurologist, it is sensitive to subtle clinical changes and can inform medical management decisions or prompt confirmatory diagnostic imaging. Its structured format enables quantitative analysis, but absolute score values do not necessarily reflect the animal’s overall neurologic well-being. The neurodisability score assigns points in a non-weighted manner. Hence, the score sum reflects the clinical status or well-being of dogs to a certain degree but comes with limitations: A mild persistent head tilt might not impair quality of life at all, whereas persistent epileptic seizures do. Relative changes in neurodisability score within individual dogs could be more important than absolute values, reflecting subtle changes in dogs’ neurologic status. However, it is an excellent measure to depict the course of neurologic deficits. For specific neurologic deficits (eg, cranial nerve impairments, gait abnormalities, mentation), sequential assessment using a score sheet is more accurate. Dividing the broader classification of neurologic deficits into mild, moderate, and severe^9^^,^^16^ allows for consistent follow-up assessments by nonspecialists, such as radiation oncologists or referring veterinarians. Despite being less detailed, this simplified grading system captures clinically relevant neurologic impairments and can be applied retrospectively, provided that a thorough clinical examination and comprehensive documentation are available. A score assessing neurologic deficits broadly is typically sufficient to capture neurologic changes and can be easily applied in clinical settings. Although advantages such as statistical interpretation of the neurodisability score are insightful, they often do not outweigh the score's simplicity and practical utility in everyday clinical practice. Interobserver disagreement may occur when applying the neurodisability score. Nevertheless, its structured approach to categorizing neurologic deficits appears to support good prospective interobserver reliability. A slightly adapted version of the score yielded an intraclass correlation coefficient of 0.83 (IQR 0.68-0.91), consistent with good agreement in prospective use.^27^

The threshold used for brain tumor progression is often a ≥ 40% volumetric increase.^20^^,^^21^ In our population, however, clinical progression was commonly observed before reaching a ≥ 40% volume increase. Only 10 clinically deteriorating dogs experienced volumetric progression ≥ 40% whereas 21 clinically and volumetrically progressive dogs did not meet the previously suggested cut-off point. In clinical studies, re-imaging typically is performed only upon corticosteroid-nonresponsive clinical deterioration and rarely repeated thereafter. This scenario is particularly relevant for intracranial tumors, where owner willingness for specific time point follow-up imaging is often limited because of the diagnostic burden, including anesthesia risks, financial constraints, and logistical challenges. Moreover, the suggested volumetric cut-off depends on prior imaging, increasing the possibility of underestimation of volumetric progression if the smallest point of tumor volume was missed. Nearly all dogs (54/59) experienced neurologic deterioration at the time of imaging, confirming progression and emphasizing the superiority of neurologic assessment.

Consistent with our findings, seizures previously have been reported as the most common complaint in dogs with intracranial tumors.^25^ Seizure control in dogs with structural epilepsy caused by brain neoplasia carries a poor prognosis when managed by medical treatment alone.^28^ The outcome improves substantially with antitumor therapy such as radiotherapy.^29^ In our findings, a large proportion of dogs (53%) presenting with seizures before treatment remained seizure-free by the end of follow-up, similar to the 44% previously reported.^29^ A subset of dogs, however, experienced delayed onset of seizure freedom or failed to achieve it entirely, with the majority of these cases diagnosed with extraparenchymal tumors. This observation suggests that tumor type, or speed of tumor response, may influence the effectiveness of radiotherapy in terms of seizure control. Furthermore, treatment guidelines for managing seizures in dogs with structural brain disease remain limited.^28^^,^^29^ In the absence of such recommendations—and given that seizure freedom was not universally achieved—it may be important to consider targeting therapeutic serum concentrations of antiseizure medications in some cases, similar to what is done in dogs with idiopathic epilepsy.

The presumed diagnosis for all extraparenchymal lesions was meningioma, whereas for all intraparenchymal lesions, glioma was the presumed tumor type. Because these were only clinical diagnoses based on imaging, other tumor types could not be excluded with certainty. Magnetic resonance imaging identifies the correct intracranial tumor type in 70% of cases.^30^ The sensitivity and specificity of conventional MRI to diagnose intracranial tumor types is 59.6% and 94.9% for extraparenchymal tumors, 84.4% and 93.7% for intraparenchymal tumors, 3% and 49% for choroid plexus tumors, and 4% and 48% for pituitary tumors, respectively.^31^ Detection of tumor infiltration is more accurate on MRI, which can lead to underestimation of tumor volume using CT.^32^ The ability to grade intraparenchymal tumors and extraparenchymal tumors based on MRI is poor.^33^^,^^34^ Stereotactic brain biopsy, on the other hand, offers a precise and minimally invasive method for diagnosing intracranial tumors, with a previously described diagnostic yield of 94.2% and should be used in the future for appropriate diagnosis.^35^^,^^36^ Because of the lack of histopathology in most cases, tumor grade could not be assessed, potentially influencing response to radiotherapy, but evidence for a differential response remains limited to human medical literature to date.^37^

In hindsight, the presumed metastatic extraparenchymal tumor located in the region of the cerebellopontine angle was more likely a choroid plexus tumor. In addition, we questioned the diagnosis of a pituitary tumor with secondary lesions. In this case, it remains unclear whether true metastases were present—a phenomenon that has been reported only very rarely—or whether the newly identified mass represented a second primary neoplasm.^38^ Furthermore, 24% of the dogs were diagnosed with concurrent spinal diseases at presentation, which could have led to misinterpretation or overestimation of the neurodisability score because of spinal ataxia in these cases. We did not track organ-specific adverse effects of medical treatment, but grades 1 and 2 adverse events have been reported in most dogs after administration of corticosteroids and anticonvulsants.^22^ If possible, dogs should be tapered rapidly from corticosteroids to minimize adverse effects of long-term corticosteroid administration.^39^

In conclusion, changes in tumor volume do not replace clinical observation. In owners with financial concerns, re-imaging can be offered with the development of medical treatment-refractory neurologic worsening, but set time points for follow-up imaging might be mostly of academic interest. Structured and regular follow-up of neurologic signs should be incorporated in a standardized manner, accompanying conventional outcome variables.

## Supplementary Material

250724_Supplementary_Neurodisabilitypaper_JVIM_aalag069

## Off-label antimicrobial declaration

The authors declare no off-label use of antimicrobials.

## Institutional animal care and use committee or other approval declaration

Approved by the Animal Ethics Council of the Canton of Zurich, Switzerland (Permit Numbers: ZH075/17, ZH021/19, and ZH202/2022).

## Abbreviations

CT: computed tomography
FLAIR: fluid-attenuated inversion recovery
GTV: gross tumor volume
IMRT: intensity-modulated radiation therapy
MRI: magnetic resonance imaging
RT: radiation therapy
T2W: T2-weighted
