# Supplementary material for: Neurologic improvement and tumor shrinkage after radiotherapy in dogs with imaging-based intracranial neoplasia
Source: J Vet Intern Med. 2026 Apr 27;40(2):aalag069. doi: 10.1093/jvimsj/aalag069 (PMC13116335; doi:10.1093/jvimsj/aalag069)
Supplement: 250724_Supplementary_Neurodisabilitypaper_JVIM_aalag069 [file 250724_supplementary_neurodisabilitypaper_jvim_aalag069.docx]

**File 1:** Neurodisability score sheet[^10^](#_ENREF_10)

|  | At dx | pre RT | post RT | @3 mo | @ 6 mo | @ 9 mo | @ 12 mo |
| --- | --- | --- | --- | --- | --- | --- | --- |
| **Date** |  |  |  |  |  |  |  |
| **Medication** |  |  |  |  |  |  |  |
| **Cranial Nerves** |  |  |  |  |  |  |  |
| PLR abnormal  Unilateral 0.5 Bilateral 1 |  |  |  |  |  |  |  |
| Menace response abnormal ^a^  Unilateral 0.5 Bilateral 1 |  |  |  |  |  |  |  |
| Strabismus 1 |  |  |  |  |  |  |  |
| Nystagmus 1 |  |  |  |  |  |  |  |
| Head tilt ^b^ 1 |  |  |  |  |  |  |  |
| Facial paralysis  Unilateral 1  Bilateral 0.5 |  |  |  |  |  |  |  |
| Absent gag reflex 1 |  |  |  |  |  |  |  |
| Dropped jaw 2 |  |  |  |  |  |  |  |
| Megaesophagus 2 |  |  |  |  |  |  |  |
| **Mentation** |  |  |  |  |  |  |  |
| Coma 1 |  |  |  |  |  |  |  |
| Obtunded 1 |  |  |  |  |  |  |  |
| Circling/head pressing 2 |  |  |  |  |  |  |  |
| Seizures Isolated 1 |  |  |  |  |  |  |  |
| Seizures Clusters 2 |  |  |  |  |  |  |  |
| Seizures Status epilepticus 3 |  |  |  |  |  |  |  |
| **Postural Responses** |  |  |  |  |  |  |  |
| Ataxic; ambulatory 1 |  |  |  |  |  |  |  |
| Ataxic; nonambulatory 2 |  |  |  |  |  |  |  |
| Unable to stand 3 |  |  |  |  |  |  |  |
| **Paresis** |  |  |  |  |  |  |  |
| Paretic; ambulatory 1 |  |  |  |  |  |  |  |
| Paretic; nonambulatory 2 |  |  |  |  |  |  |  |
| Plegia 3 |  |  |  |  |  |  |  |
| Total score |  |  |  |  |  |  |  |

^a^ Not scored if PLR also abnormal.

^b^ Not scored if nystagmus also present.
